# RE: A guideline for the inpatient care of children with pyelonephritis

**DOI:** 10.4103/0256-4947.75792

**Published:** 2011

**Authors:** Kamal Akl

**Affiliations:** Associate Professor of Pediatrics Consultant Pediatric Nephrologist Jordan University Hospital College of Medicine University of Jordan Pobox 831373 Amman 11183-Jordan T: +96265514458 F: +9624644710 kachbl@yahoo.com

**To the Editor:** We read with great interest the article by Chishti et al,^1^ and would like to congratulate the authors for developing these timely guidelines. However, the authors mentioned that febrile urinary tract infection associated renal scarring is highest in the first year of life. For infants less than one year of age, it would be more appropriate to use the term renal parenchymal defect instead of scarring, as recommended by the NICE guidelines.^2^ Parenchymal defects discovered in infancy are usually the result of renal dysplasia rather than acquired scars. Renal dysplasia occurs when the developing kidney is subjected to an in utero insult, including vesicoureteral reflux, at a critical period of nephrogenesis.^3^ Along the same lines, in the dysplastic kidney, recurrent febrile urinary tract infections add insult to injury, thus speeding up kidney function deterioration.^4^

Authors of original article declined to respond.
